# Novel Bone Void Filling Cement Compositions Based on Shell Nacre and Siloxane Methacrylate Resin: Development and Characterization

**DOI:** 10.3390/bioengineering10070752

**Published:** 2023-06-23

**Authors:** Bridget Jeyatha Wilson, Lizymol Philipose Pampadykandathil

**Affiliations:** Division of Dental Products, Department of Biomaterial Science and Technology, Biomedical Technology Wing, Sree Chitra Tirunal Institute for Medical Sciences and Technology, Thiruvananthapuram 695 012, India; jeyatha21@gmail.com

**Keywords:** shell nacre, ormocer, bone void filling cement, bone defects

## Abstract

Shell nacre from *Pinctada* species has been extensively researched for managing bone defects. However, there is a gap in the research regarding using shell nacre powder as a cement with improved biological and physicochemical properties. To address this, bone void filling cement was formulated by incorporating shell nacre powder and an organically modified ceramic resin (ormocer). The shell nacre powder was specifically processed from the shells of *Pinctada fucata* and analysed using thermogravimetric analysis (TGA), X-ray diffraction spectroscopy, Fourier transform infrared (FTIR), and Raman spectroscopy, confirming the presence of organic constituents and inorganic aragonite. Trace element analysis confirmed the eligibility of shell nacre powder for biomedical applications. Next, the ormocer SNLSM2 was synthesized through a modified sol–gel method. FTIR, Raman, TGA, and transmission electron microscopy studies revealed the presence of a ladder-structured siloxane backbone and methacrylate side chain. To develop chemical curable composite shell nacre cement (SNC), different amounts of shell nacre (24%, 48%, and 72%) were added to the SNLSM2 resin, and the impact on the physicochemical properties of the cement was studied. Among the compositions, SNC 72 exhibited significantly lower linear polymerization shrinkage (0.4%) and higher compressive (>100 MPa) and flexural strength (>35 MPa). SNC 72 was radiopaque, and the exotherm generated during the cement curing was minimal. Cytotoxicity studies with L929 cells revealed the non-cytotoxic nature of the cement. Overall, the findings of this study prove that the shell nacre cement is a promising candidate for managing bone voids.

## 1. Introduction

The management of bone defects depends on factors such as the size, shape, and location of the defect, as well as the age and overall health of the patient. The main aim of managing metaphyseal defects is to provide mechanical support for the joint surface and restore bone stock. Irregular bone defects that are less than 5 mm or between 5 and 10 mm in size are treated with either bone cement or bone cement with screw augmentation [1,2,3]. Polymethyl methacrylate (PMMA) cement is the most studied and clinically used material for the management of fragility fractures [4], vertebral compression fractures [5], revision arthroplasty defects [3], bone voids after infection [6]/tumour resections [7], etc. Apart from its inert application in arthroplasty, it is used for bone defect management, solely for its mechanical properties, regardless of the potential toxicity of the monomer, volumetric shrinkage, or the exotherm generated during polymerization [8,9,10].

Methyl methacrylate (MMA) and PMMA powder are the primary constituents of PMMA cement. In order to address the issues of polymerization shrinkage and exotherm generation, several organic matrices or combinations of organic matrices such as urethane dimethacrylate, poly(ethylene) glycol dimethacrylate, triethylene glycol dimethacrylate (TEGDMA), bisphenol-A-glycidyl methacrylate (Bis GMA) [11,12,13,14], terpolymer (bisphenol-A-ethoxy methacrylate, Bis GMA, TEGDMA) [15], acrylic acid and styrene [16], etc., have been investigated with the aim of replacing MMA. However, despite these efforts, the problems of exotherm generation, toxicity, and polymerization shrinkage have not been completely addressed. Consequently, there is a need for an alternative resin matrix to overcome these challenges.

Ormocer is a three-dimensional multifunctional inorganic–organic hybrid resin with an inorganic siloxane backbone instead of an organic carbon backbone. It is easily synthesized by a modified sol–gel method using methacrylate-substituted alkoxysilanes through the process of hydrolysis and condensation to form the inorganic siloxane network, whereas the methacrylate moieties remain intact [17]. It has been widely investigated in the field of dental cements due to its non-cytotoxic nature, low polymerization shrinkage, and superior mechanical properties [18,19,20,21], particularly the photocured cured composites of ladder-structured siloxane methacrylate resin [22]. However, there is no report of usage of this resin for orthopaedic applications to our knowledge. So, considering the low shrinkage properties, mechanical properties, and non-cytotoxicity, we have selected the ormocer resin strategy.

The next issue to address is the inertness of PMMA cement, which prevents it from bonding to the implanted bone. To overcome this limitation, with the concept of bioactivity, many researchers have modified the cement formula with silanated glass [11], apatite wollastonite glass ceramic (AW-GC) [23], nano-sized titania particles [24], hydroxyapatite (HA) [14], strontium-substituted HA [13], HA with BMP-2 [25], synthetic combeite glass-ceramic particles, barium boro aluminosilicate glass and silica particles [26], borosilicate glass [27], graphene oxide [28], etc. Similarly, bioactive self-setting cements such as calcium phosphate, calcium sulphate, tricalcium phosphate, calcium silicate, and magnesium phosphate cements have also been investigated. However, these cements often exhibit brittleness, poor mechanical properties, and faster resorption, which may not provide the long-term mechanical support required [29,30,31,32,33,34,35,36]. Additionally, bone cements based on oyster, clam, and abalone shell powder have been studied, but none of these cements exhibited the required mechanical properties [37,38,39,40]. Hence, the unsatisfactory biological performance of PMMA-based cements and the weaker mechanical performance of the modified cements emphasizes the critical need for an alternative cement that can offer both biological and physicochemical properties together.

Shell nacre/mother of pearl is the inner nacreous layer of pearl oyster shells composed of calcium carbonate crystals arranged in aragonite form with an organic layer inter-tiled between them in a brick-and-mortar fashion. It was Camprasse et al. who first developed an artificial dental root made of shell nacre (Bioracine) and the Lopez group who first revealed the in vitro osteogenesis of shell nacre without any inducer [41,42,43]. An in vivo comparison study proved that shell nacre induced new bone formation whereas PMMA caused necrosis and reduced bone mineralization [44]. Moreover, osteogenesis, radiopacity, osseo-integration, high fracture toughness and strength, biodegradability, anti-osteoporotic activity, and angiogenesis properties have proven shell nacre as a suitable biomaterial for hard tissue applications [45,46,47,48,49]. Although shell nacre powder has been made as a putty by mixing with autologous blood [50,51], there is no processing method to set and mould shell nacre powder into defined anatomical forms with suitable mechanical properties.

Therefore, the aim of this study is to develop shell nacre cement with desirable characteristics such as low polymerization shrinkage, minimal exotherm, excellent mechanical properties, and non-cytotoxicity. Firstly, the processing of *Pinctada fucata* shells was carried out to obtain shell nacre powder, which was characterized using scanning electron microscopy (SEM), FTIR, Raman, TGA, XRD, and optical emission spectroscopy with inductively coupled plasma (OES-ICP). Next, shell nacre-containing ladder-structured siloxane methacrylate resins were synthesized and characterized by FTIR, Raman, TGA, and TEM. Finally, the SNC was formulated by combining the synthesized SNLSM2 resin with shell nacre powder at different concentrations (24%, 48%, and 72%) along with other additives. The resulting cement compositions were assessed for radiopacity, linear polymerization shrinkage, and mechanical properties. The exothermic behaviour of the cement was studied by isothermal DSC, and the cytotoxicity of the cement was studied with L929 cells. Through these comprehensive evaluations, this study aims to provide valuable insights into the physicochemical properties of shell nacre cement, highlighting the synergistic action of shell nacre powder and ormocer resin.

## 2. Materials and Methods

### 2.1. Collection of Shells and Removal of Outer Prismatic Layer

**Figure 1:**
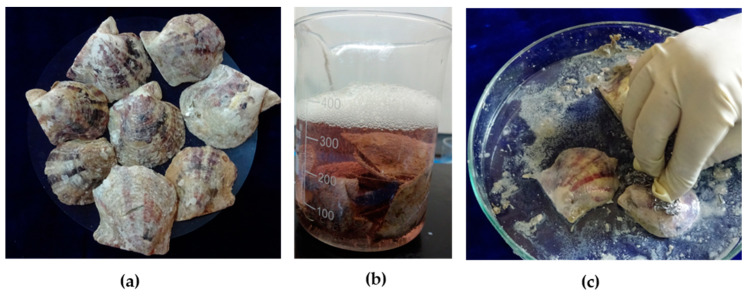
Processing of pearl oyster shells: (**a**) shells of *Pinctada fucata*; (**b**) soaking of shells in acetic acid and NaCl solution; and (**c**) scrubbing the outer prismatic layer.

*Pinctada fucata* shells (Figure 1a) that had been discarded after fishing activities were collected from the Kayalpattinam coast of the Gulf of Mannar. The shells were washed in running water, and the external impurities such as tunicates and deposited dirt were removed mechanically. This was followed by an intensive detergent wash, and shells were dried at 37 °C. Dried shells were soaked in 15–20% acetic acid with 5%NaCl (Figure 1b). After 1 h of soaking, the external prismatic layer was scrubbed off (Figure 1c). Shells were sonicated in distilled water to remove the acid impurities, and then dried nacreous shells were observed by SEM (FEI Quanta 200, Eindhoven, The Netherlands).

#### 2.1.1. Processing of Shell Nacre Powder

Nacreous shells were broken into small pieces, powdered using a planetary ball mill (Retsch, Haan, Germany), and sieved using a vibrational sieve (Retsch, Germany). Shell nacre powder was collected after being passed through a 20 µm sieve and used for the synthesis of resin and as filler for shell nacre cement formulation.

#### 2.1.2. Characterization of Shell Nacre Powder

The particle size and morphology of shell nacre powder were studied by SEM. Shell nacre powder was mixed with potassium bromide at a ratio of 1:400, and the FTIR spectrum was recorded from 400 to 3750 cm^−1^ at a resolution of 4 cm^−1^ (FTIR Carry 600, Agilent technologies, Bangalore, India). The Raman spectrum of shell nacre powder was recorded using confocal Raman microscopy (alpha 300RA, Witec, Ulm, Germany) with a 532 nm laser. The XRD spectrum was measured (Bruker, D8 Advance, Germany) in the 2θ range of 10–70° with CuKά radiation in the incremental step of 5°, and peaks were identified using the JCPDS (Joint Committee on Powder Diffraction Standards) database. TGA analysis of shell nacre powder was carried out using SDT-2960, TA Instruments, heating the powder from room temperature to 1000 °C (heating rate of 10 °C/min) in a nitrogen atmosphere. Trace elements such as Cu, Fe, Mg, Mn, Zn, Cd, Pb, Hg, and Se were estimated using optical emission spectroscopy with inductively coupled plasma (OES-ICP) (PerkinElmer, Hopkinton, MA, USA).

### 2.2. Synthesis of SNLSM1 and SNLSM2

The first step in the synthesis was hydrolysis of the precursor 3-trimethoxysilyl propyl methacrylate (3-TMSPM) (Sigma-Aldrich, St. Louis, MO, USA) with deionized water at a ratio of 1:3. After 30 min of hydrolysis, 6N NaOH, 1% shell nacre powder (1 wt.% of 3-TMSPM), and diethyl ether were added, and the reaction was continued for 8 h to synthesize SNLSM1 resin. The same procedure was followed for SNLSM2 resin with 2% shell nacre powder (2 wt.% of 3-TMSPM). Later, the reaction mix was washed and separated with distilled water and diethyl ether to remove the alkali. Then, the ether phase containing the resin was collected, evaporated, and dried at 37 °C until the complete removal of water.

### 2.3. Characterization of Resins

The refractive index of the resin was observed using an Abbey refractometer (ATAGO 3T, Tokyo, Japan). The FTIR spectra of the synthesized resins were measured using FTIR Carry 600 (Agilent Technologies). The spectra were obtained from 600 to 4000 cm^−1^ with a resolution of 4 cm^−1^. A thick layer of resin was applied on a glass slide, and the Raman spectrum was recorded using confocal Raman microscopy. Thermal stability of the resins (SNLSM1, SNLSM2) was evaluated by heating from room temperature to 1000 °C at the heating rate of 10 °C/min, in a nitrogen atmosphere. The SNLSM2 resin (in methanol) was drop-casted onto formvar-coated 200 mesh copper grids (EM sciences, Hatfield, PA, USA) and allowed to dry overnight. The grid was then observed by using a transmission electron microscope (TEM) (Hitachi H-7650 Tokyo, Japan) at an accelerated voltage of 80 kV. All graphs were drawn using Origin Pro (version 8.5). 

### 2.4. Formulation of Shell Nacre Cements (SNC 24/48/72)

**Figure 2:**
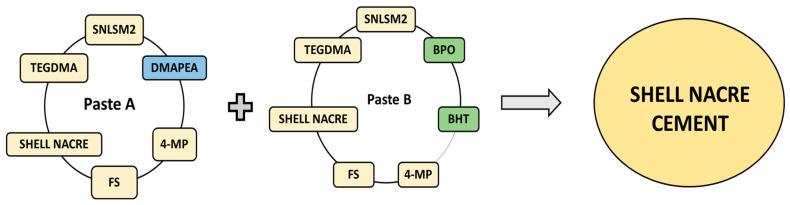
Representation of the formulation of Paste A and Paste B, which were mixed together to prepare the shell nacre cement. DMAPEA (blue coloured) is present only in Paste A. BPO and BHT (green coloured) are present only in Paste B, whereas the other ingredients are common to both pastes.

Shell nacre cement was formulated as a two-paste system (Pastes A and B) with the shell nacre powder and the synthesized SNLSM2. Paste A included SNLSM2 (12%), triethylene glycol dimethacrylate (TEGDMA) (12%) (Sigma-Aldrich), dimethyl amino phenyl ethanol (DMAPEA) (0.4%) (Sigma-Aldrich, MO, USA), fumed silica (FS) (3%), traces of 4-methoxy phenol (4-MP) (Sigma-Aldrich, MO, USA), and shell nacre powder (24%/48%/72%). Paste B included SNLSM2 (12%), TEGDMA (12%), benzoyl peroxide (BPO) (0.7%) (Merck, Darmstadt, Germany), traces of butylated hydroxytoluene (BHT) (Merck, Darmstadt, Germany) and 4-MP, FS (3%), and shell nacre powder (24%/48%/72%) (Figure 2). All the percentages mentioned are weight percentage and indicated as (wt.%).

#### Preparation of Shell Nacre Cements (SNC 24/48/72)

**Figure 3:**
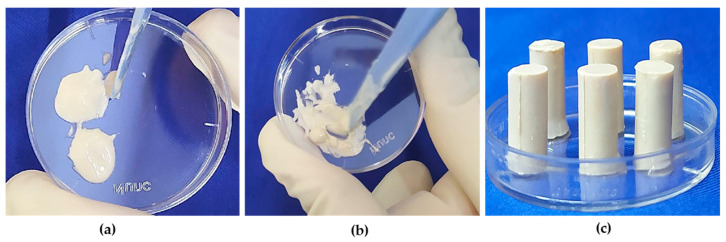
Preparation of shell nacre cement samples: (**a**) equal amounts of Paste A and Paste B; (**b**) mixing of Paste A and Paste B; (**c**) after mixing, the paste was filled into the compressive strength (CS) mould, and CS samples were released after curing.

The first step in the preparation of Paste A was thinning of SNLSM2 with TEGDMA to form the resin matrix. DMAPEA was added next, followed by 4-MP, which were allowed to dissolve completely in the resin matrix. Subsequently, FS was added and mixed well. Finally, shell nacre powder (24%/48%/72%) was added slowly and mixed thoroughly to form Paste A. Similarly, Paste B was prepared in the same way, in which BPO, BHT, and 4-MP were added to the resin matrix and completely dissolved. Afterwards, FS was added, and lastly, shell nacre powder (24%/48%/72%) was added to form Paste B. Equal amounts of Paste A (SNC 24/48/72) and Paste B (SNC 24/48/72) were mixed for 30 sec by hand spatulation (Figure 3a,b). The time before it started to set was recorded as the working time, and the time required for the complete setting of the cement (which was understood by the non-sticky nature of the cement) was noted as the setting time. Further, cured samples of specific dimensions (Figure 3c) were prepared according to the needs of experiments.

### 2.5. Characterization of Shell Nacre Cement

#### 2.5.1. Radiopacity Evaluation

The radiopacity of the cement was studied based on the ASTM standard F640 2012 [52]. Scout images of SNC samples (6 mm diameter and 3 mm height) and the reference material, an aluminium step wedge (aluminium alloy EN 1050 containing 99.5% Al), were acquired using microcomputed tomography (µCT Scanco 40, Bruttisellen, Switzerland). Using ImageJ software, the grayscale intensity of the samples was analysed (n = 6). A standard curve was plotted with the thickness of the Al wedge (0.5, 1, 1.5, 2, 2.5, and 3 mm) against the mean grayscale intensity of the Al wedge. Radiopacity of the cement samples equivalent to the thickness of the Al wedge (mm) was determined from the standard curve.

#### 2.5.2. Evaluation of Linear Polymerization Shrinkage (LPS)

The percentage linear polymerization shrinkage was evaluated as previously described [18]. In brief, shell nacre cement paste was packed in a 6 mm diameter and 3 mm height stainless steel mould and allowed to cure. After 30 min, the sample was released from the mould, and the internal diameter of the mould was measured accurately using a digital calliper with an accuracy of 0.01 mm (Mitutoyo, Kawasaki, Japan). The diameter of the cured sample was measured at six points in all directions, and the mean value was calculated. The measurement was repeated for six samples, and the percentage linear polymerization shrinkage was calculated using the formula LPS (%) = (internal diameter of the mould − sample diameter)/internal diameter of the mould) × 100.

#### 2.5.3. Evaluation of Mechanical Properties

Compressive strength samples were prepared using a brass mould of 3 mm diameter and 6 mm depth. The mould was placed on a strip of transparent sheet on a metal plate, and the shell nacre cement pastes were packed into the mould. The second strip of transparent sheet was placed on the top followed by a second metal plate. The mould and strip of film between the metal plates were pressed to displace excess material and allowed to cure for 30 min. Similarly, flexural strength test specimens were prepared using a mould of 25 mm length, 2 mm depth, and 2 mm thickness. The cured samples were removed from the mould and incubated in distilled water at 37 °C for 24 h and 22 °C for 1 h before testing. Using the corresponding jigs, compressive strength and flexural strength were determined using a universal testing machine (Model 1011, Instron, High Wycombe, UK) with crosshead speeds of 5 mm/min and 1 mm/min, respectively.

#### 2.5.4. Investigation of Exotherm Generated

Exotherm generation of the curing SNC 72 cement was studied by isothermal DSC (Universal V4.5A, TA Instruments Inc., New Castle, DE, USA) at 24 °C and 37 °C for 30 min, and the enthalpy change (δH) was recorded.

#### 2.5.5. Cytotoxicity Studies

##### Direct Contact Study

A cytotoxicity test of the sterile SNC 72 cement sample was performed based on ISO 10993-5 [53]. The positive control used was a stabilized polyvinylchloride (PVC) disc, and the negative control was ultra-high molecular weight polyethylene (UHMWPE). All the samples were sterile, and the detailed procedure of ethylene oxide sterilization is described in the Supplementary Materials. L929 mouse fibroblast cells (ATCC) were cultured in Dulbecco’s minimal essential medium (DMEM; Gibco, New York, NY, USA) supplemented with 10% fetal bovine serum (FBS; Gibco, USA) and 1% penicillin streptomycin (Gibco, USA). Cells were seeded in a 24-well plate at a density of 3 × 10^4^ cells/well, incubated at 37 °C with 5% CO_2_, and allowed to grow until sub-confluency. Three replicates of SNC 72 and the controls were placed on the monolayer of L929 cells. After 24 h contact with the material, the cell monolayer was observed under a phase contrast microscope for the response around the materials. Qualitative evaluation based on changes in cell morphology, zone of lysis, vacuolization, detachment, and membrane disintegration was graded on a scale of 0 (none), 1 (slight), 2 (mild), 3 (moderate), and 4 (severe).

##### Cell Viability Assay

Cell viability of SNC 72 after 24 h, 48 h, and 72 h of curing was studied with L929 cells. The detailed procedure is provided in the Supplementary Materials.

### 2.6. Statistical Analysis

All statistical analyses were performed using GraphPad Prism (version 9.5.1). An ordinary one-way ANOVA, along with Tukey’s multiple comparison test, was performed to compare the linear polymerization shrinkage and compressive and flexural strength of SNC 24, SNC 48, and SNC 72. Differences were considered statistically significant only if *p* ≤ 0.05, and the symbols indicated were ns—*p* > 0.05, *—*p* ≤ 0.05, **—*p* < 0.01, ***—*p* < 0.001, and ****—*p* < 0.0001. The data are presented as the mean and the standard deviation of the mean.

## 3. Results and Discussion

Shell nacre is a superior composite material. So far, only the organic matrix of shell nacre and shell nacre pieces or powder have been studied to demonstrate the osteogenic potential. Although it is commercially available as a bone substitute in powder form/pieces/osteosynthesis devices [54], there is a lack of research in which shell nacre from *Pinctada fucata* is exploited for bone defect management. Another major hurdle is that there is no clearly demonstrated processing method to obtain shell nacre powder with both organic and inorganic parts. This study introduces two novel aspects. First, the shell nacre powder used in the study was comprised of both organic and inorganic constituents and specifically processed from the shells of *Pinctada fucata*. Second, shell nacre-containing ladder-structured siloxane methacrylate resin was used to prepare the in situ curing composite cement. This unique composition has not been reported in previous studies, which utilized oyster powder (unnamed) [37], nacre powder (unspecified) [38], clam shell powder [39], or whole abalone shell powder [40] for composite cement preparation. Therefore, to our knowledge, this is the first bone void filling cement of its kind.

### 3.1. Processing of Shells

**Figure 4:**
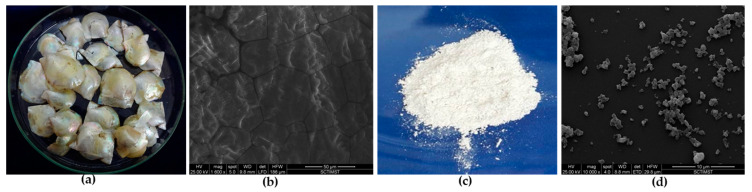
Processing of shells: (**a**) nacreous shells after prismatic layer removal; (**b**) SEM observation of nacreous shells; (**c**) macroscopic appearance; and (**d**) SEM observation of shell nacre powder.

The first and foremost task of the present work was to process the shells of *Pinctada fucata* and to remove the outer prismatic layer. Since the objective of the present study was to obtain shell nacre powder with both an inorganic matrix and an organic matrix together, use of a mild acetic acid along with NaCl was attempted. This method enabled the faster removal of calcite with brisk effervescence, and further, manual scrubbing easily removed the outer prismatic layer. Subsequently, the lustrous nacreous shells (Figure 4a) were washed and dried. SEM observation of lustrous nacreous shells exhibited the characteristic brick-and-mortar structure of shell nacre (Figure 4b) where the aragonite crystals were glued together with the organic matrix, as seen by Sun and Bhushan [55]. The lustrous nacreous shells were ball milled and sieved to obtain shell nacre powder (Figure 4c). SEM analysis of the shell powder revealed an irregular morphology (Figure 4d). The obtained shell nacre powder was heterogenous with particle size smaller than 0.5 µm (ImageJ analysis). In contrast, other studies have reported and used shell nacre powder of larger sizes, such as 50–100 µm [50] and 42.5 µm [51], for implantation studies.

### 3.2. Characterization of Shell Nacre Powder

**Figure 5:**
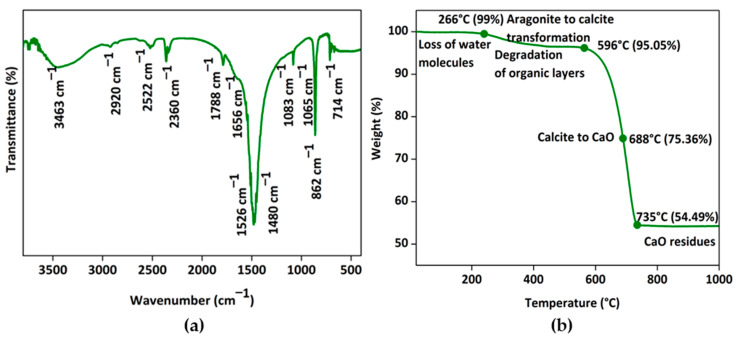
Characterization of shell nacre powder: (**a**) FTIR spectrum from 400 to 3750 cm^−1^ and (**b**) thermogram from RT to 1000 °C confirming the presence of both organic and inorganic constituents.

The obtained shell nacre powder was characterized by FTIR, TGA, Raman, XRD, and ICP-OES analysis. FTIR analysis of shell nacre powder is shown in Figure 5a. The presence of peaks for the internal vibration modes of CO_3_^2−^ ions of calcium at 714 cm^−1^, 862 cm^−1^, 1083 cm^−1^, and 1480 cm^−1^ and a splitting peak at 714 cm^−1^ confirmed the aragonite form. The strongest peak of the spectrum was at 1480 cm^−1^, which was due to the overlap of peaks of the organic matrix and carbonate ions. The broad peak at 3463 cm^−1^ was the stretching modes of OH/NH of the organic matrix or the adsorbed water molecules as reported [56,57,58]. A strong peak at 2920 cm^−1^ was attributed to the CH stretching modes of the organic matrix. The peaks of OH of HCO_3_-groups in the crystal lattice or at the mineral/organic interface groups of carboxylic groups were observed at 2522 cm^−1^, as well as stretching of carbonyl groups of acidic proteins at 1788 cm^−1^. Peaks at 1656 and 1526 cm^−1^ were attributed to the amide I/amide II bonds of proteins of the organic matrix. A shoulder at 1065 cm^−1^ was assigned to the C-O stretching of carbohydrates present in the organic matrix; further, the peak also indicated the SO_3_-vibration of the sulphated glycosaminoglycans. Similar observations were recorded during the investigation of organic polymers of Venus clams [59]. It was reported that the organic matrix of shell nacre is comprised of the water-soluble matrix and the water-insoluble matrix. The water-soluble matrix contains the acidic proteins and polysaccharides, whereas the water-insoluble matrix contains the chitin, lipid, and the alanine and glycine rich silk-like proteins [60]. In the present study, the peaks of aragonite, proteins, carbohydrates, and water were observed, and the results were consistent with the findings of Balmain et al. and Zouari et al. during investigations of the organic content of the shell nacre of *Pinctada maxima* shells [56] and *Pinctada radiata* shells [61]. Thus, the FTIR study confirmed the presence of both organic and inorganic constituents in shell nacre powder.

The thermogram of shell nacre powder is shown in Figure 5b. The initial loss of water content was observed at 266 °C, which was followed by the degradation of organic content and the transformation of the aragonite to calcite at 596 °C. The gradual major loss (41%) occurred between 596 °C and 735 °C during the conversion of calcite to calcium oxide with the release of CO_2_, leaving a final residue of calcium oxide at 1000 °C. As organic molecules are complexed with aragonite, the degradation of organic molecules occurred only at 596 °C. Once the organic content was lost, the aragonite crystal was transformed into calcite, and gradually the entire calcite was transformed into calcium oxide. Thus, the thermogravimetry changes proved that shell nacre powder was comprised of 5% organic and 95% inorganic content. Similar TGA results were reported by Balmain et al. and Zouari et al. during investigations of the organic content of the shell nacre of *Pinctada maxima* shells [56] and *Pinctada radiata* shells [61].

**Figure 6:**
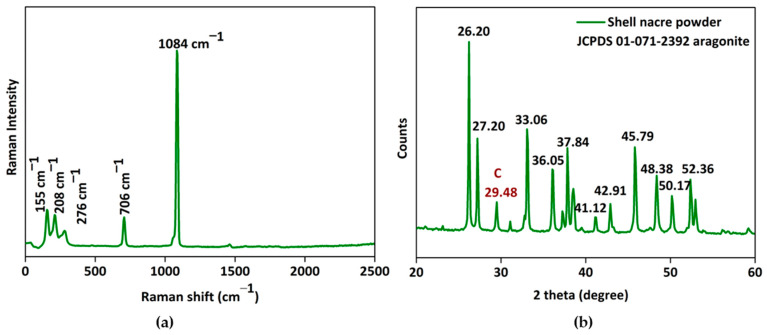
Characterization of shell nacre powder: (**a**) micro-Raman spectrum and (**b**) XRD pattern confirming the aragonite nature of shell nacre powder.

The micro-Raman spectrum (Figure 6a) exhibited the characteristic carbonate stretching vibrations of aragonite at 1084 cm^−1^ and lattice vibration modes of aragonite at 155 cm^−1^, 208 cm^−1^, and 706 cm^−1^. The X-ray diffraction pattern (Figure 6b) of shell nacre powder matched with the aragonite crystals of JCPDS 01-071-2392 and had characteristic sharp diffraction lines of well-crystallized aragonite of single mineral phase. Both the Raman and XRD studies proved that the shell nacre powder contained an aragonite form of calcium carbonate. However, a calcite peak at 276 cm^−1^ in the Raman spectrum and a calcite diffraction line at 29.48° in the XRD spectrum were seen, which may be due to the heat generated during the milling of shell nacre powder, due to traces of the external prismatic layer, or due to the presence of calcite in shell nacre itself [56,61,62].

Table 1 presents the result of the trace element analysis of shell nacre powder. Copper was not detected, while manganese and zinc were present at very low levels (0.772 and 0.4827 ppm, respectively). Iron was detected at 5.889 ppm, and magnesium was found at a concentration of 102.87 ppm. Among the deleterious heavy metals, cadmium and selenium were below the detection limit, while lead and mercury were present at the levels of 1.4 ppm and 0.772 ppm, respectively. Further, the trace element analysis was concluded based on ASTM F2103-18 [63] and ASTM F1609-08 [64], which specify that the permissible limits of mercury and lead should be less than 5 and 30 ppm, respectively, and the total content of harmful heavy metals should be less than 50 ppm. In the present study, shell nacre powder contained mercury and lead, which were below the permissible limits, and the other heavy metals cadmium, selenium, and copper were below the detection limits. The total content of harmful heavy metals in the shell nacre powder was below 50 ppm. Therefore, the shell nacre powder obtained through the acetic acid-based method is suitable for use in biomedical applications, as it contains both organic and inorganic contents and is free of harmful heavy metals.

### 3.3. Characterization of Resins

The next objective was synthesis and characterization of the shell nacre-containing siloxane methacrylate resins. During the synthesis, alkoxy silane underwent hydrolysis, NaOH favoured the condensation of the hydrolysed precursor, and a ladder-structured inorganic siloxane network with methacrylate side chain was formed. The synthesized resins showed no change in refractive index ~1.47 after the addition of 1% and 2% shell nacre powder. Similar refractive index values were recorded for ormoresins [65], zirconium-containing resin [21], and strontium-containing resin [20].

**Figure 7:**
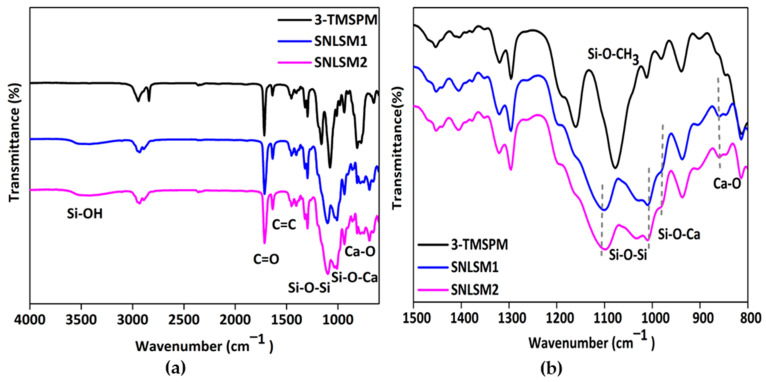
FTIR analysis of resins: (**a**) FTIR spectrum from 600 to 4000 cm^−1^ and (**b**) FTIR spectrum from 800 to 1500 cm^−1^ showing the presence of bimodal shaped peaks.

The molecular architecture of the siloxane was identified by FTIR analysis, which reveals distinct peaks in the 1000–1200 cm^−1^ region. A random structure is characterized by multiple peaks, a cage structure by a single sharp peak, and a ladder structure by bimodal peaks [66]. FTIR analysis (Figure 7a) of the resins proved the presence of bimodal peaks at 1101 cm^−1^ and 1033 cm^−1^. This confirmed the polycondensation and formation of a ladder-structured inorganic siloxane backbone. It was reported that an alkali catalyst favoured the formation of ladder-structured siloxane, and the results were in agreement with previous studies [22,67,68]. A shift in the peak from 980 cm^−1^ to 987 cm^−1^ in both the resins confirmed the presence of Si-O-Ca in the network. Further, the presence of a peak at 862 cm^−1^ (Figure 7b) corresponded to Ca-O in the siloxane network of SNLSM1 and SNLSM2, and the findings were consistent with previous studies [69]. Both the resins retained the characteristic acrylate groups C=C at 1637 cm^−1^ and C = O at 1716 cm^−1^. Thus, the FTIR analysis confirmed that the formation of shell nacre integrated a ladder-structured siloxane skeleton with methacrylate side chain.

**Figure 8:**
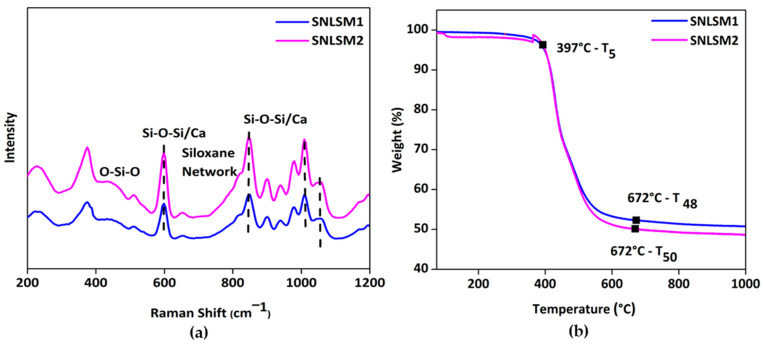
Characterization of resins: (**a**) micro-Raman spectrum showing the formation of four-membered siloxane units and (**b**) thermogravimetric analysis confirming the intense siloxane backbone with shell nacre integration.

Raman analysis of the resins (Figure 8a) proved that the siloxane unit was four-membered, which was confirmed by the presence of a peak at 598 cm^−1^, and similar results were documented in a previous study [70]. Further, siloxane network formation was understood by a peak around 829 cm^−1^ as reported by the Artaki group [71]. Again, the integration of shell nacre was confirmed by the shift in siloxane peaks at 598, 846, 1008, and 1058 cm^−1^ in both the resins, and these findings were consistent with previous research [72].

Figure 8b shows the thermogravimetric analysis of the resins from 25 °C to 1000 °C. T_5_ of both SNLSM1 and SNLSM2 was recorded at around 397 °C. SNLSM2 exhibited T_50_ (temperature at which 50% degradation occurred) at 672 °C, whereas no T_50_ was noted for SNLSM1, and it was left with ~52% residue. Previous studies proved that ladder-structured siloxane methacrylate has an intense siloxane network [68] and high thermal stability [73]. TGA results of SNLSM1 were similar to an earlier study of ladder-structured siloxane methacrylate, which was also left with ~52% residue [22]. This revealed that the addition of shell nacre had not modified the siloxane network of SNLSM1, whereas in the case of SNLSM2, shell nacre modified the siloxane network. Being a divalent, calcium modified the organic silica network, and the growth of siloxane was prevented after the integration of shell nacre [69].

**Figure 9:**
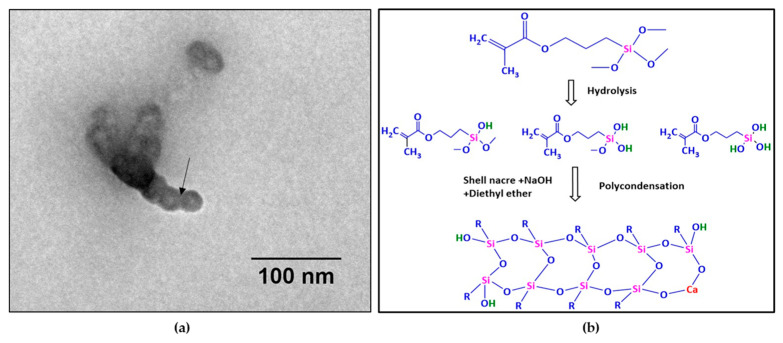
(**a**) TEM image of SNLSM2 resin (scale bar represents 100 nm); (**b**) scheme of synthesis of SNLSM2 resin.

Further, TEM observation of SNLSM2 (Figure 9a) revealed the ladder-structured siloxane backbone of the resin. The four-membered siloxane unit was seen as small dots with interconnections between them. The interconnection was labelled with an arrow mark. The size of each siloxane unit was ~10 nm (ImageJ). The ladder structure of SNLSM2 was smaller when compared with the previously reported ladder structure of LSM [22]. This reflected the change in the structure of SNLSM2 (Figure 9b) after the addition of shell nacre. As SNLSM2 evidenced the presence of shell nacre in all the above characterization studies, it was further taken for the cement formulation.

### 3.4. Formulation of Shell Nacre Cement

Control of setting time, faster mixing, and enhanced mechanical properties are the advantages of the two-paste system. SNLSM2 (12%) was diluted with an equivalent amount of triethylene glycol dimethacrylate (12%) and formulated as a two-paste system with initiator, activator, stabilizers, fumed silica, and shell nacre powder. During the mixing of Paste A and Paste B, BPO along with DMAPEA promoted the polymerization of the organic network by breaking the C=C bond of the methacrylate moieties of the resin and TEGDMA, and the chain growth was continued. The amounts of BPO, DMAPEA, and BHT were optimized, and the working and setting times of the cement were 3 min and 6 min (Figure S1), respectively. The two pastes were mixed easily, and samples of specific dimensions were prepared according to the needs of experiments.

### 3.5. Characterization of Shell Nacre Cement

There is a need for a bone void filling cement with synergistic properties such as radiopacity, mechanical properties, non-cytotoxicity, low shrinkage, and minimal exotherm. To achieve this synergism, either the organic resin matrix or the filler part can be modified. In the case of our SNC cement, both strategies were employed by modifying the organic resin matrix with the ormocer SNLSM2 and introducing shell nacre powder in the filler part. Different cements were prepared by varying the amount of shell nacre powder (24%, 48%, and 72%), and the effect on the physicochemical properties such as radiopacity and LPS (%) and the mechanical properties of the cement was studied.

#### 3.5.1. Radiopacity Evaluation

Radiopacity is essential for the successful clinical monitoring of the material. Barium sulphate and zirconium dioxide are used as radiopacifier in the clinically available PMMA cement. However, their toxicity and effects on mechanical properties demand an alternative [74]. In the present study, shell nacre powder acted as a radiopacifier. The radiopacity values of SNC 24, SNC 48, and SNC 72 were 1.7, 2.3, and 2.9 mm equivalent to the thickness of the Al wedge (Figure S2). The radiopacity of the cement composition was increased with a higher concentration of shell nacre, and the inherent radiopacity of shell nacre conferred the cement compositions with radiopacity. Previous studies have reported that many other radiopacifier, such as triphenyl bismuth, tantalum powder, bismuth salicylate, iodine-containing co-polymers, bromine-containing co-polymer [74], and gold [75], were investigated as radiopacifiers of bone cement. However, none of them have been proved to be as osteogenic and multi-functional as shell nacre powder.

#### 3.5.2. Evaluation of LPS

**Figure 10:**
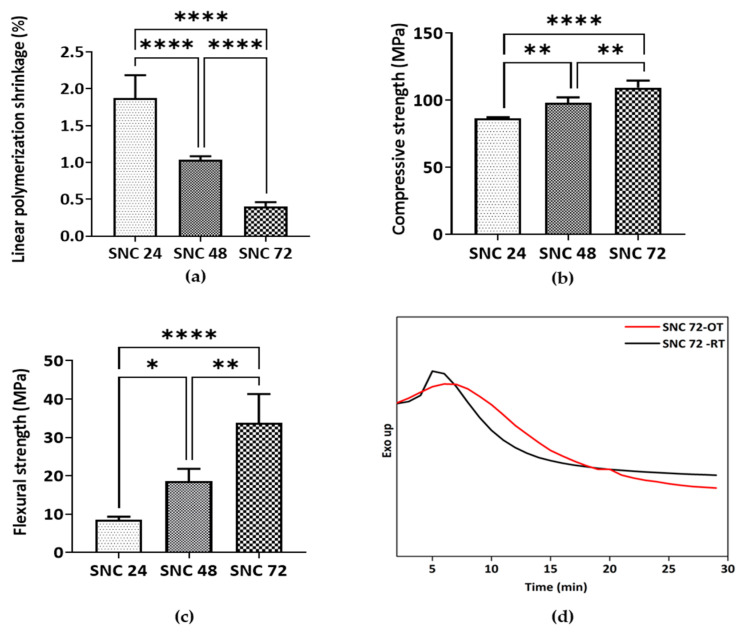
Characterization of shell nacre cement: (**a**) Evaluation of linear polymerization shrinkage (n = 6); (**b**) compressive strength (n = 4); and (**c**) flexural strength (n = 4) analysis of cured SNC 24, SNC 48, and SNC 72 samples. Ordinary one-way ANOVA. Tukey’s multiple comparisons test. *—*p* < 0.05, **—*p* < 0.01, ****—*p* < 0.0001. Results are shown with mean and standard deviation of the mean. (**d**) Isothermal DSC of SNC 72 at 24 °C (SNC 72-OT) and 37 °C (SNC 72-RT) showing the exotherm generated during the curing of SNC 72.

The LPS (%) value of the compositions SNC 24, SNC 48, and SNC 72 was 1.8, 1.03, and 0.4% (Figure 10a), respectively. This indicated a reduction in the shrinkage with an increasing amount of shell nacre powder. Shrinkage of cement after polymerization is a major concern, and commercially available bone cements typically have a range of 5–10% shrinkage [76]. In contrast, the shrinkage of SNC 72 was only 0.4%, which was significantly lower than the other cements. However, even the minimal shell nacre powder-containing cement SNC 24 had a value of 1.8%, which reflected the multi-functionality of SNLSM2. Therefore, the synergistic action of both SNLSM2 and shell nacre reduced the linear polymerization shrinkage of the SNC compositions.

#### 3.5.3. Evaluation of Mechanical Properties

SNC 72 exhibited average compressive and flexural strength of ~110 MPa (Figure 10b) and ~35 MPa (Figure 10c), respectively, which is significantly higher than the other cement compositions. Being an inorganic–organic hybrid filler, shell nacre was incorporated into the cement compositions without any silanization. Both the ormocer SNLSM2 and shell nacre contributed to the mechanical properties, which was understood by the increase in both compressive and flexural strength with increasing shell nacre content. Previous studies by Shen et al. and Du et al. reported that the compressive strength of calcium sulphate composites of oyster powder and abalone shell powder was only ~11 MPa [37] and ~5 MPa [40], respectively. Another study with calcium phosphate cement showed that an increasing concentration of nacre reduced the compressive strength [38]. In contrast, the compressive strength of the current SNC 72 composite was ~110 MPa, which was 10–20 times higher than the reported calcium sulphate composites, and the mechanical properties were enhanced with increasing shell nacre content. A previous study by Wu et al. reported that addition of amphiphilic raspberry particles to PMMA reduced the compressive strength [77], and similarly, addition of magnesium oxide also reduced the mechanical properties of cement [78]. However, in the shell nacre cement compositions, the mechanical properties were improved by the bonding of shell nacre powder with the SNLSM2 resin. A study by Yang et al. found that use of acrylic acid and styrene instead of methyl methacrylate in PMMA cement made the compressive strength lower than that of PMMA cement [16]. In contrast, SNLSM2 imparted good compressive strength even to the cement with a low concentration of shell nacre powder (24 wt.%).

#### 3.5.4. Investigation of Exotherm Generated

SNC 72 exhibited better mechanical properties, radiopacity, and low linear polymerization shrinkage, and so SNC 72 was selected for further studies (isothermal DSC and cytotoxicity). As curing is affected by temperature, both the room temperature of the surgery room and the human body temperature were considered, and isothermal DSC was carried out at both 24 °C and 37 °C. The exotherm generated by SNC 72 was 2.227 J/g and 0.5781 J/g at 37 °C and 24 °C, respectively (Figure 10d). Although the heating rate and other conditions were similar, variations in enthalpy change δH were found owing to curing of SNC 72 at two different temperatures. A previous study reported that the exotherm generated by PMMA cement was 52 kJ/mole of PMMA, which was very high and was another major drawback of PMMA cement [10]. Another study reported no significant change in maximum polymerization temperature of Palacos cement after modification with 2-hydroxyethyl methacrylate, calcium chloride, and sodium carbonate [78]. Similarly, addition of graphene and graphene oxide powders to PMMA showed no changes in exotherm generation [79]. In contrast, the combination of shell nacre powder and SNLSM2 resulted in a reduction in the exotherm in the present study, which was very minimal when compared with the commercially available cements and other experimental cements.

### 3.6. Cytotoxicity Studies

**Figure 11:**
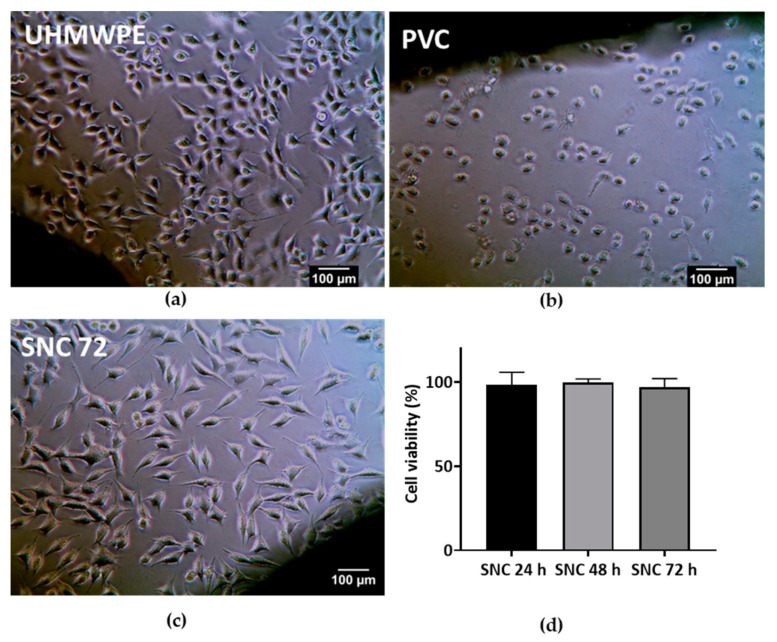
Cytotoxicity studies: Direct contact of (**a**) SNC 72; (**b**) negative control UHMWPE; and (**c**) positive control PVC with L929 cells for 24 h. Magnification 20 X. Scale bar represents 100 µm. (**d**) Cell viability assay of SNC 72 cement samples after 24, 48, and 72 h of curing with L929 cells.

The cytotoxicity evaluation of SNC 72 was conducted in accordance with ISO 10993-5 using mouse fibroblast cell line L929. L929 cells exhibited no features of cytotoxicity, after contact negative control UHMWPE (Figure 11a), whereas the positive control PVC, showed severe cytotoxicity (Figure 11b) as expected. During contact with the cured sterile SNC 72 cement sample, L929 cells maintained the spindle morphology and no cytotoxicity features were observed as shown in Figure 11c. Both SNC 72 and the negative control were graded 0, and the positive control was graded 4. Cell viability of SNC 72 samples after 24 h, 48 h, and 72 h of curing was studied with L929 cells. After 24 h contact with samples, L929 cells showed more than 95% cell viability (Figure 11d). This again confirmed the non-cytotoxic nature of the SNC 72 samples. A previous study found that addition of increasing amounts of calcium sulphate (40% by weight) to PMMA cement resulted in a reduction in cell viability [80]. On the other hand, a higher addition of shell nacre (72% by weight) to SNC 72 did not cause any toxicity issues.

In summary, *Pinctada fucata* shells were processed to obtain shell nacre powder, which consisted of 5% organic component and 95% inorganic aragonite. Further, the synthesized SNLSM2, which featured shell nacre, integrated a ladder-structured siloxane backbone and methacrylate side chain. Shell nacre cements were then formulated using different concentrations of shell nacre powder (24%, 48%, and 72%) and SNLSM2 (12%). Among the formulated cements, SNC 72 was selected for its low linear polymerization shrinkage (%), high radiopacity, and good mechanical properties. Importantly, SNC 72 was non-cytotoxic and exhibited a minimal exotherm. Overall, this study demonstrates the successful development of shell nacre cement and its advantageous properties.

## 4. Conclusions and Future Perspectives

SNC 72 is a pioneering bone void filling cement made of shell nacre powder and ladder-structured siloxane methacrylate resin. It surpasses the commercially available options and the other experimental cements due to its superior properties such as low linear polymerization shrinkage, minimal exotherm generation, good mechanical properties, and non-cytotoxic nature. Taken together, the findings of this study prove that SNC 72 has the potential to become a leading bone void filling cement for bone defect management. With its unique composition and impressive properties, this cement could offer significant advantages over existing options on the market.

Further research and testing will be necessary to fully explore its potential and refine its formula for clinical use. Future investigations into in vitro osteogenesis with bone marrow mesenchymal stem cells, in vivo long-term degradation studies, preclinical evaluation based on the ISO 10993-1 standard, and in vivo bone void studies in osteoporotic models will provide even more evidence for the superiority of this cement. Additionally, examining the flow properties and formulating an injectable cement will enhance the versatility of the cement.

## 5. Patents

1. Lizymol Philiphose Pampadykandathil, Bridget Jeyatha Wilson, and Venkiteswaran Kalliyanakrishnan. 2018. A process for the synthesis of shell nacre containing bio-resin for dental and orthopedic applications. Indian patent No. 400578. Date of Grant: 30 June 2022.
2. Lizymol Philiphose Pampadykandathil and Bridget Jeyatha Wilson. 2020. Low-cost bioactive bone cement. Indian patent No. 410827. Date of Grant: 2 November 2022.

## Figures and Tables

**Table 1:** ICP-OES analysis of shell nacre powder.

Elements Analysed	Total Amount (ppm)
Cu	BDL ^1^
Mn	0.772
Zn	0.4827
Fe	5.889
Mg	102.87
Cd	BDL ^1^
Pb	0.772
Hg	1.4
Se	BDL ^1^

^1^ BDL: Below the lower detection limit for Cu (0.0097 ppm), Cd (0.0027 ppm,) Se (0.0750 ppm).

## Data Availability

The data used to support the findings of this study are available from the corresponding author upon reasonable request.

## Supplementary Materials

The following supporting information can be downloaded at: https://www.mdpi.com/article/10.3390/bioengineering10070752/s1, Materials and methods of ethylene oxide sterilization process and cell viability [81], Figure S1: Working and setting time of the formulated cement, Figure S2: Radiopacity evaluation.
