# Supplementary material for: Novel Bone Void Filling Cement Compositions Based on Shell Nacre and Siloxane Methacrylate Resin: Development and Characterization
Source: Bioengineering (Basel). 2023 Jun 23;10(7):752. doi: 10.3390/bioengineering10070752 (PMC10376770; doi:10.3390/bioengineering10070752)
Supplement: Supplementary file 1 [file bioengineering-10-00752-s001.zip › bioengineering-2333400-supplementary.pdf]

bioengineering

MDPI

Supplementary data of

Novel Bone Void Filling Cement Compositions Based on Shell Nacre and Siloxane Methacrylate Resin: Development and Characterization

Bridget Jeyatha Wilson and Lizymol Philipose Pampadykandathil *

Division of Dental Products, Department of Biomaterial Science and Technology, Biomedical Technology Wing, Sree Chitra Tirunal Institute for Medical Sciences and Technology, Kerala 695 012 India; jeyatha21@gmail.com
* Correspondence: lizymol@sctimst.ac.in

Materials & Methods

Ethylene oxide (EO) sterilization process

Sterilization of cement samples were performed in an in-house EO sterilizer Sterivac (3M Healthcare, MN, USA) employing 100% ethylene oxide gas dedicated for sterilizing biomaterials and biomedical devices components. The sterilization system operates on procedures validated as per ISO 11135: Sterilization of health care products [1]. SNC 72 samples were packed in standard Tyvek bags suitable for EO sterilization. Sterilization was performed at 37°C and maintained a minimum of 550 mg/L (corresponding to a pressure rise of 325 mbar) EO gas using a 170 g EO canister in 227-liter chamber. EO exposure was given for 180 minutes. Three air purge cycles followed by 3 h aeration was done prior to removal of load from the sterilization chamber for ensuring removal of residual gases.

Cell viability

$3x10^4$ L929 cells were seeded per well in 24 well plates and incubated at 37±1°C, 5% CO_2. After monolayer (sub-confluent) formation, replenished with fresh medium and test samples SNC 24h, SNC 48h and SNC 72h were placed on the cells. After 24 h incubation, the medium was removed and 1 ml of 2% Alamar medium (Alamar reagent + DMEM medium) was added to each well. After 3h incubation, it was read at 570 nm. The cell viability (%) was determined using the formula; Cell viability (%) = [(O2 x A1) - (O1 x A2)/ (O2 x P1) - (O1 xP2)] *100 where, O1- molar extinction coefficient (E) of oxidized Alamar blue at 570 nm = 80586, O2- E of oxidized Alamar blue at 600 nm = 117216, A1-the absorbance of test wells at 570 nm, A2-the absorbance of test wells at 600 nm, P1-the absorbance of cell control at 570 nm, P2-the absorbance of cell control at 600 nm.

Results and discussion

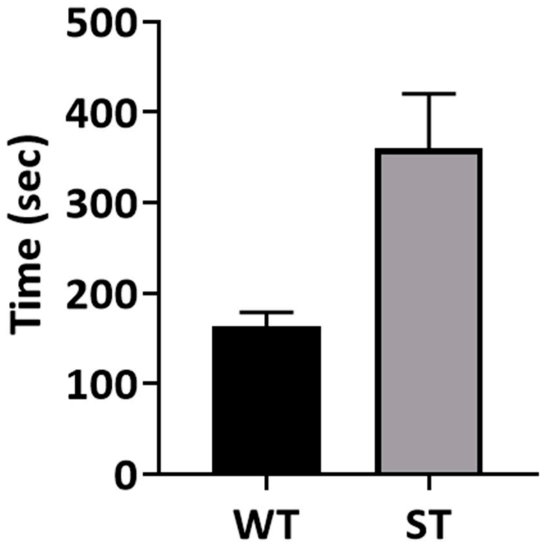

Figure S1. Working and setting time of the formulated cement

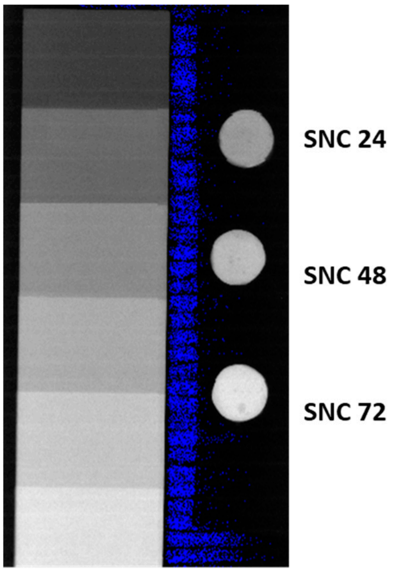

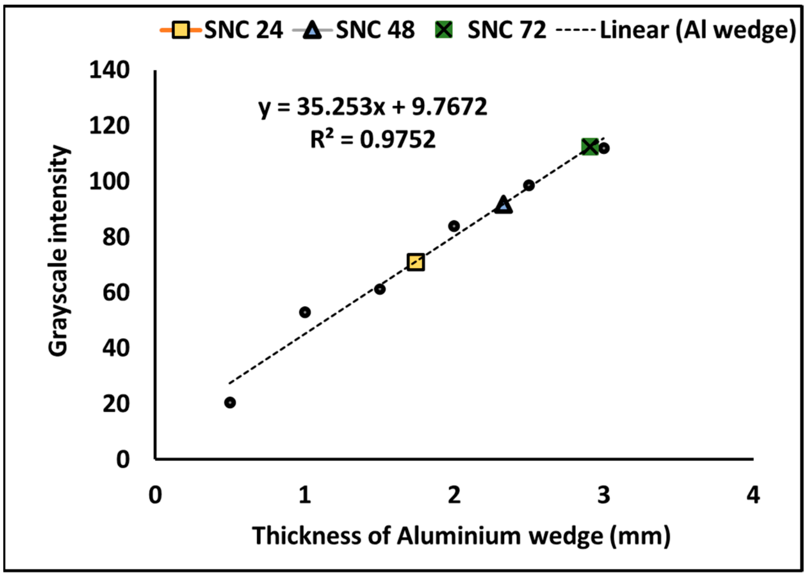

(a) **(b)**

Figure S2. Radiopacity evaluation: **(a)**Scout images of shell nacre cement SNC 24, 48 and 72 with Aluminum wedge obtained using micro-computed tomography; **(b)** A standard curve was plotted with the thickness of each step of the Al wedge against the mean grayscale intensity of the Al step wedge. The radiopacity of the SNC samples equivalent to the thickness of the Al wedge was determined from the standard curve.

References

1. *ISO 11135:2014*; Sterilization of Health-Care Products—Ethylene Oxide—Requirements for the Development, Validation and Routine Control of a Sterilization Process for Medical Devices. Available online: https://www.iso.org/standard/56137.html (accessed on 15 May 2023).
